# Polynomial Perceptrons for Compact, Robust, and Interpretable Machine Learning Models

**DOI:** 10.3390/e28040453

**Published:** 2026-04-15

**Authors:** Edwin Aldana-Bobadilla, Alejandro Molina-Villegas, Juan Cesar-Hernandez, Mario Garza-Fabre

**Affiliations:** 1 Cinvestav, Unidad Tamaulipas, Ciudad Victoria 87130, Mexico; edwyn.aldana@cinvestav.mx (E.A.-B.); jcesar.hernandez@cinvestav.mx (J.C.-H.); mario.garza@cinvestav.mx (M.G.-F.); 2 Secihti—Centro de Investigación en Ciencias de Información Geoespacial, Scientific and Technological Park of Yucatan, Merida 97302, Mexico

**Keywords:** Polynomial Perceptrons, resource-efficient machine learning, model interpretability, explainable AI

## Abstract

This paper introduces the Polynomial Perceptron (PP), a structured extension of the classical perceptron that incorporates explicit polynomial feature expansions to model nonlinear interactions while preserving analytical transparency. By expressing feature interactions in closed functional form, PP captures higher-order dependencies through a compact set of learned coefficients, establishing a principled trade-off between expressivity and parameter efficiency. The proposed architecture is evaluated across heterogeneous domains, including text, image, and structured data tasks, under controlled experimental settings with parameter-matched baselines. Performance is assessed using standard metrics such as classification accuracy and model complexity (parameter count). Empirical results demonstrate that low-degree PP models achieve competitive accuracy compared to multilayer perceptrons and convolutional neural networks, while requiring significantly fewer parameters. An ablation study further analyzes the impact of polynomial degree on predictive performance, revealing diminishing returns beyond moderate degrees and highlighting favorable efficiency–accuracy trade-offs. A key advantage of PP lies in its intrinsic interpretability. Unlike conventional deep learning models that rely on post hhoc explanation methods, PP provides direct analytical insight through its explicit polynomial structure, enabling decomposition of predictions into feature-, token-, or patch-level contributions without surrogate approximations. Overall, the results indicate that PP offers a lightweight, interpretable, and computationally efficient alternative to standard neural architectures, particularly well-suited for resource-constrained environments and applications where transparency is critical.

## 1. Introduction

The rapid expansion of artificial intelligence (AI) has produced models capable of solving increasingly complex tasks with unprecedented accuracy. Contemporary machine learning systems have demonstrated remarkable performance across language processing, computer vision, and structured prediction problems. However, these advances have largely been driven by scaling model size and computational resources. Prominent examples, such as DeepSeek [1] and GPT-based models [2], illustrate that predictive gains are often accompanied by massive parameterization and substantial infrastructure requirements.

While deep architectures are flexible and expressive, their effectiveness is frequently tied to scale rather than structural efficiency. This “parameter proliferation” increases energy consumption and environmental impact [3], raises barriers to entry for research and deployment, and concentrates AI development within a limited set of well-resourced institutions. Beyond computational considerations, another fundamental limitation concerns interpretability. The hierarchical and highly nonlinear transformations within deep networks obscure the relationship between inputs and outputs, making it difficult to trace how individual features or interactions influence a prediction.

Although the field of explainable AI (XAI) has proposed numerous techniques to mitigate this opacity, most approaches rely on post hoc explanations, such as surrogate models, saliency maps, or local approximations, that are external to the learned function and may not faithfully reflect the true internal decision process [4]. Consequently, predictive performance is often achieved at the expense of transparency, a trade-off that is particularly problematic in high-stakes domains including healthcare, finance, and public policy. These challenges highlight the need for alternative modeling that reconciles nonlinear expressivity, parameter efficiency, and intrinsic interpretability. In this context, explicitly structured models, where feature interactions are represented in closed functional form, offer a promising direction. By maintaining analytical traceability of learned coefficients while enabling higher-order interactions, compact polynomial formulations provide a principled pathway toward machine learning systems that are both expressive and inherently transparent.

The proposed framework employs bounded-degree polynomial expansions over input representations to capture nonlinear feature interactions while preserving analytical transparency and controlled model complexity. By limiting the degree of expansion, the approach achieves a principled balance between expressive power and parameter efficiency, alleviating the scalability challenges typically associated with high-dimensional nonlinear models. Central to the proposal is a coefficient-based attribution mechanism that directly quantifies the contribution of individual features, tokens, or localized components to model predictions. In contrast to post hoc interpretability techniques.

The proposed formulation is closely related to Functional Link Neural Networks (FLNNs) [5,6] and Higher-Order Neural Networks (HONNs) [7], both of which rely on explicit nonlinear feature expansions followed by linear parameterization. However, the present approach differs in its architectural organization and scope of application. Classical FLNN and HONN formulations typically assume a global expansion over the full input space, which leads to rapid combinatorial growth in the number of terms as dimensionality increases. In contrast, as detailed in the experiments, our proposal introduces structured, bounded-degree polynomial constructions with localized interactions, allowing the model to scale to higher-dimensional inputs (e.g., images) through spatial decomposition. Additionally, while prior work on FLNNs and HONNs has primarily focused on function approximation, control, or signal processing tasks, this work evaluates such constructions under modern optimization settings and across heterogeneous data modalities (text, image, and structured data), with an emphasis on the trade-offs between locality, degree, and coefficient growth.

For clarity, we refer to this structured formulation as a Polynomial Perceptron, emphasizing its interpretation as a single-layer model with explicit polynomial feature mappings and learned linear coefficients under bounded-degree constraints. The remainder of this paper is organized as follows: Section 2 discusses the relevant literature. Section 3 introduces the necessary background and theoretical foundation. Section 3.2 describes Polynomial Perceptrons, while Section 4 presents their evaluation across several use cases. Finally, Section 6 summarizes our main findings and highlights potential pathways for future research.

## 2. Related Work

The use of explicit polynomial feature interactions has a long and well-established history in machine learning and neural computation. Early work on HONNs demonstrated that multiplicative interactions among inputs significantly enhance representational capacity beyond linear perceptrons [7]. Notably, Sigma–Pi networks introduced neurons that compute weighted sums of input products, enabling compact representations of nonlinear decision boundaries [5,6]. These architectures formalized the idea that polynomial interactions can be embedded directly within neural units rather than constructed through deep compositional stacking. Closely related are FLNNs, which expand the input space through deterministic basis functions (commonly polynomial or trigonometric expansions) followed by linear adaptive learning [8]. FLNNs showed that shallow architectures with enriched input representations can approximate complex nonlinear mappings without hidden layers, foreshadowing modern discussions on expressivity versus depth.

From a statistical modeling standpoint, explicit polynomial interactions are foundational in polynomial regression and generalized linear models (GLMs) with interaction terms, where nonlinearities are introduced through manually specified higher-order terms [9]. In these frameworks, models remain linear in parameters while nonlinear in features, a principle shared with higher-order neural formulations. An alternative strategy for handling polynomial interactions is found in kernel methods, particularly support vector machines using polynomial kernels [10]. The kernel trick implicitly computes high-dimensional polynomial feature maps without explicitly constructing interaction terms. While computationally efficient, this implicit representation obscures individual interaction coefficients, limiting direct interpretability. Subsequent work on polynomial networks and tensor-based multiplicative architectures extended these ideas by factorizing higher-order interactions or stacking multiplicative layers [11].

In parallel, the literature on explainable AI (XAI) has focused largely on post hoc interpretability techniques, including surrogate modeling and local approximations [4]. These methods attempt to explain complex models externally, rather than embedding interpretability within the predictive structure itself. However, most XAI methods rely on *post hoc* strategies to generate explanations for the outputs of *black-box* models. *Post hoc* techniques include feature attribution methods, such as gradient-based, perturbation-based, and concept-based approaches [12,13].

Gradient-based explanation techniques aim to interpret machine learning models (especially deep neural networks) by analyzing how small changes in the input affect the model’s output. The central idea is to compute the output gradient associated with the input features, to quantify the sensitivity of the model’s prediction to each of them. More formally, given a trained model $f(\overset{\to }{x})$, with $\overset{\to }{x}\in R^{k}$, the gradient $\nabla_{\overset{\to }{x}}f\left(\overset{\to }{x}\right)$ represents the vector of partial derivatives of the output for each of the *k* input features. A high gradient value for a specific feature indicates that small perturbations in that feature would cause significant changes in the output, suggesting a strong influence or relevance. These methods are useful for high-dimensional and opaque models, such as deep neural networks, where interpretability is limited. By visualizing or analyzing these gradients, one can identify which parts of the input contribute most to the decision-making process. Several refinements and variants have been proposed to improve the stability and clarity of gradient-based explanations, including *Saliency Maps* [14], *Integrated Gradients* [15], and *Gradient-Weighted Class Activation Mapping* (Grad-CAM) [16].

Perturbation-based explanation methods aim to understand model predictions by observing how changes in the input affect the output. Unlike gradient-based approaches, which rely on internal derivatives, perturbation methods treat the model as a black box and infer feature importance through systematic input modifications. Specifically, these approaches assess the contribution of each feature $x_{i}\in \overset{\to }{x}$ by measuring the change in prediction $f(\overset{\to }{x})$ when $x_{i}$ is altered. The importance of $x_{i}$ is expressed as Δi=f(x→)−f(x→xi), where x→xi is the input vector with feature $x_{i}$ removed, masked, or replaced by a baseline value. These approaches are model-agnostic and can be applied to any predictive system, making them particularly attractive for auditing black-box models. Perturbation-based methods include *Local Interpretable Model-agnostic Explanations* (LIME) [17], which fits an interpretable surrogate model to local perturbations of the input; *SHapley Additive exPlanations* (SHAP) [18], which builds on cooperative game theory to compute fair attribution scores for each feature; and *Occlusion Sensitivity* [19], commonly used in computer vision by occluding parts of the input image and measuring the effect on the output. These methods tend to be more intuitive to interpret than gradients, especially when derivatives are unstable or poorly defined. However, they are computationally more expensive, as they require repeated evaluation of the model on modified inputs.

Concept-based explanation methods aim to bridge the gap between abstract model representations and human-understandable concepts by analyzing internal activations of deep neural networks (DNNs). These approaches do not explain decisions from raw input features, but instead use high-level semantic concepts defined by humans. One of the most prominent methods is *Testing with Concept Activation Vectors* (TCAV) [20], which evaluates the sensitivity of a model’s prediction to a user-defined concept by projecting internal activations onto a direction (vector) corresponding to that concept. Let $f:\overset{\to }{x}\to y$ be a trained DNN, and let $h_{l}\left(\overset{\to }{x}\right)$ be the activation of layer *l* for input $\overset{\to }{x}$. Given a set of examples representing a concept, a linear classifier (e.g., SVM) is trained to distinguish between this concept and random examples. The resulting hyperplane defines a *concept activation vector* (CAV), ${\overset{\to }{v}}_{C}$, in the activation space. The directional derivative of the model output *y* with respect ${\overset{\to }{v}}_{C}$ quantifies the so-called *concept sensitivity*. A high value of concept sensitivity indicates that concept *C* is important for the prediction of $\overset{\to }{x}$. *Concept Activation Regions* (CAR) [21] extended this idea by identifying localized sub-networks or groups of neurons responsible for encoding semantic concepts. CAR combines concept discovery with network dissection to automatically extract and evaluate regions that contribute meaningfully to concept activation, improving the interpretability of high-dimensional layers. Concept-based approaches enhance the interpretability of DNNs by grounding explanations in user-relevant abstractions, rather than low-level features. They are especially valuable in domains where understanding the presence and influence of meaningful concepts is critical (e.g., medicine, law, fairness).

As such, there is growing interest in revisiting foundational learning formulations as a means to explore models that are structurally transparent, compact, and amenable to interpretation. This perspective motivates the examination of architectural paradigms in which interpretability and analytical tractability arise directly from the model formulation, rather than being recovered through post hoc analysis.

In this context, the following sections begin with a concise review of the classical perceptron as a linear reference model. We then describe a structured extension based on bounded-degree polynomial feature mappings, which we refer to as a Polynomial Perceptron (PP) for convenience. This formulation is subsequently analyzed and evaluated empirically across representative tasks, with the goal of illustrating its behavior under different input representations and architectural configurations.

## 3. Theoretical Foundation

In this section, we outline the fundamental theoretical principles that underpin our work. We begin by revisiting the conventional perceptron, introduced by Rosenblatt [22,23], which models a linear decision boundary using a thresholded weighted sum of inputs. While powerful for linearly separable tasks, the model’s simplicity renders it inadequate for problem spaces that deviate from such a structure. To transcend this constraint, non-linear perceptrons introduce non-linear transformations that enable the modeling of complex, non-linear functions that enrich representational power by capturing feature interactions via higher-order terms—a strategy that elegantly blends expressiveness with interpretability. Starting from this foundation, we illustrate how they can be composed into richer architectures—schematically visualized—to address diverse tasks beyond linear classification.

### 3.1. Perceptron

In 1958, at the Cornell Aeronautical Laboratory, Frank Rosenblatt developed a generalization of the *McCulloch-Pitts* neuron model, expressed as follows:

(1) $$\;u\left(\overset{\to }{x}\right)=w_{0}+w_{1}x_{1}+w_{2}x_{2}+\ldots +w_{k}x_{k}\;,$$

where the weights $w_{i}$ induce a hyperplane that defines a linear decision boundary, enabling the inference of an output value for the input vector $\overset{\to }{x}\in R^{k}$, with $w_{0}$ serving as the bias term. The optimal weights are obtained through a learning process that aims to minimize a predefined loss function, quantifying the discrepancy between the model’s output $u(\overset{\to }{x})$ and the corresponding ground truth $t_{\overset{\to }{x}}$. Given a dataset D composed of tuples of the form $\left(\overset{\to }{x},t_{\overset{\to }{x}}\right)$, the loss function is defined as follows:

$$L=∥t_{\overset{\to }{x}}-u\left(\overset{\to }{x}\right){∥}_{\forall (\overset{\to }{x},t_{\overset{\to }{x}})\in D}\;,$$

where ∥·∥ denotes a norm, commonly instantiated as *Mean Squared Error* (MSE), *Mean Absolute Error* (MAE), or *Categorical Cross Entropy* (CE). Optimization algorithms, such as *Stochastic Gradient Descent* (SGD), are employed to minimize this loss function. These methods update the weights iteratively based on the gradient of the loss function concerning each weight parameter:

$$w_{j}\leftarrow w_{j}-\eta \frac{\partial L}{\partial w_{j}}\;,$$

where η>0 is the learning rate, and $\frac{\partial L}{\partial w_{j}}$ is the partial derivative of the loss with respect to the weight $w_{j}$. Through repeated updates across multiple training epochs, the weights converge to values that (locally) minimize the loss function, optimizing the model’s performance on the task. For illustration, if MSE is used as the loss function, the partial derivative of L with respect to $w_{i}$ is expressed as follows:

(2) $$\frac{\partial L}{\partial w_{i}}=\sum_{(\overset{\to }{x},t_{\overset{\to }{x}})\in D}\left(t_{\overset{\to }{x}}-u(\overset{\to }{x})\right)x_{i}\;.$$

The perceptron represents a fundamental milestone in computer science and mathematical modeling. It is capable of generalizing relationships in a wide variety of practical problems. However, it has a significant limitation. In 1969, Marvin Minsky and Seymour A. Papert published the seminal work *Perceptrons* [24], in which they rigorously demonstrated that the simple perceptron—along with similar models such as ADALINE—can only classify linearly separable data. This limitation exposed the inherent inability of such models to learn non-linear decision boundaries, which ultimately fueled the development of multilayer architectures and the modern era of deep learning.

### 3.2. Polynomial Perceptrons

A *Polynomial Perceptron* (PP) extends the basic perceptron to model non-linearly separable problems by introducing terms that capture interactions among the components of a feature vector $\overset{\to }{x}\in R^{k}$. We define a PP as a neural processing unit $u(\overset{\to }{x})$ which transforms $\overset{\to }{x}$ into a sum of terms of the form $w_{ij\ldots r}x_{1}^{i}x_{2}^{j}\cdots x_{k}^{r}$, under the following assumptions:

1. The coefficient $w_{ij\ldots r}\in R$ represents the weight of a non-additive interaction of $x_{1}^{i},x_{2}^{j},\ldots ,x_{k}^{r}$.
2. The degree of each term is the sum of the exponents of all *x* components in that term.
3. The degree of $u(\overset{\to }{x})$, denoted as *d*, is the maximum degree among its terms.
4. For every term $w_{ij\ldots r}x_{1}^{i}x_{2}^{j}\cdots x_{k}^{r}$ of any $u(\overset{\to }{x})$ of degree *d*, it holds that i+j+⋯+r≤d.

As an illustration, consider $\overset{\to }{x}=\left[x_{1},x_{2}\right]$. Let $u_{1}\left(\overset{\to }{x}\right)$ and $u_{2}\left(\overset{\to }{x}\right)$ be two neural units of degree d=2 and d=3, respectively, defined as follows:

(3) $$u_{1}\left(\overset{\to }{x}\right)=w_{00}+w_{01}x_{2}+w_{02}x_{2}^{2}+w_{10}x_{1}+w_{11}x_{1}x_{2}+w_{20}x_{1}^{2}\;,$$

(4) $$u_{2}\left(\overset{\to }{x}\right)=w_{00}+w_{10}x_{1}+w_{01}x_{2}+w_{20}x_{1}^{2}+w_{11}x_{1}x_{2}+w_{02}x_{2}^{2}+w_{30}x_{1}^{3}+w_{21}x_{1}^{2}x_{2}+w_{12}x_{1}x_{2}^{2}+w_{03}x_{2}^{3}\;.$$

Both $u_{1}\left(\overset{\to }{x}\right)$ and $u_{2}\left(\overset{\to }{x}\right)$ satisfy the four conditions defined above. Moreover, note that the number of terms in $u(\overset{\to }{x})$ depends on *d* and *k*, and is given by:

(5) $$terms\left(d,k\right)=\frac{(d+k)!}{d!\;k!}\;.$$

Applying Equation (5) to the above example, we obtain $\frac{4!}{2!2!}=6$ and $\frac{5!}{3!2!}=10$, which matches the actual number of terms in Equations (3) and (4), respectively. In general, $u(\overset{\to }{x})$ can be expressed as follows:

(6) $$u\left(\overset{\to }{x}\right)=w_{00\ldots 0}+w_{10\ldots 0}x_{1}+w_{01\ldots 0}x_{2}+\cdots +w_{00\ldots 1}x_{k}+\cdots +w_{d0\ldots 0}x_{1}^{d}+w_{0d\ldots 0}x_{2}^{d}+\cdots +w_{00\ldots d}x_{k}^{d}\;.$$

**Figure 1:**
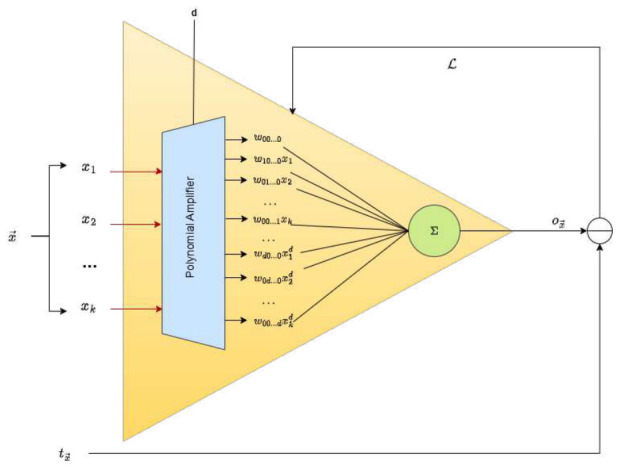
Schematic diagram of a polynomial unit.

Based on Equation (6), a schematic representation of $u(\overset{\to }{x})$ is shown in Figure 1. When an input instance $\overset{\to }{x}=\left[x_{1},x_{2},\ldots ,x_{k}\right]$ is processed, it is passed through what we define as a *polynomial amplifier*, a computational unit that expands the components $x_{i}$ into higher-degree polynomial terms. These terms are then added to produce an output value $o_{\overset{\to }{x}}$, which approximates a target value $t_{\overset{\to }{x}}$. As with linear units, this approximation results in a loss L, which can be minimized iteratively by adjusting each weight $w_{ij\ldots r}$ in the direction of the negative derivative of L with respect to $w_{ij\ldots r}$.

### 3.3. Learning Process for a Polynomial Perceptron

Similar to linear units, the learning process for $u(\overset{\to }{x})$ is performed over a dataset D containing multiple tuples ($\overset{\to }{x},t_{\overset{\to }{x}})$. Assuming MSE as the loss function, the discrepancy L between the ground truth value $t_{\overset{\to }{x}}$ and the model output $u(\overset{\to }{x})$ can be quantified as follows:

(7) $$L=\frac{1}{2}\sum_{(\overset{\to }{x},t_{\overset{\to }{x}})\in D}{\left(t_{\overset{\to }{x}}-u\left(\overset{\to }{x}\right)\right)}^{2}\;.$$

As previously noted, L is minimized by iteratively updating the weights $w_{ij\ldots r}$ using the following rule:

(8) $$w_{ij\ldots r}=w_{ij\ldots r}+\Delta w_{ij\ldots r}\;,$$

where $\Delta w_{ij\ldots r}$ represents the adjustment in the direction of the negative gradient of L with respect to $w_{ij\ldots r}$, and is given by a reformulation of the delta rule according to Equation (9):

(9) $$\Delta w_{ij\ldots r}=-\eta \frac{\partial L}{\partial w_{ij\ldots r}}\;.$$

The gradient term is derived as follows:

(10) $$\frac{\partial L}{\partial w_{ij\ldots r}}=\frac{\partial }{\partial w_{ij\ldots r}}\left(\frac{1}{2}\sum_{(\overset{\to }{x},t_{\overset{\to }{x}})\in D}{\left(t_{\overset{\to }{x}}-u\left(\overset{\to }{x}\right)\right)}^{2}\right)\;,$$

(11) $$\frac{\partial L}{\partial w_{ij\ldots r}}=\frac{1}{2}\sum_{(\overset{\to }{x},t_{\overset{\to }{x}})\in D}2\left(t_{\overset{\to }{x}}-u\left(\overset{\to }{x}\right)\right)\frac{\partial (t_{\overset{\to }{x}}-u\left(\overset{\to }{x}\right))}{\partial w_{ij\ldots r}}\;,$$

(12) $$\frac{\partial L}{\partial w_{ij\ldots r}}=\sum_{(\overset{\to }{x},t_{\overset{\to }{x}})\in D}\left(t_{\overset{\to }{x}}-u\left(\overset{\to }{x}\right)\right)\frac{\partial (t_{\overset{\to }{x}}-u\left(\overset{\to }{x}\right))}{\partial w_{ij\ldots r}}\;.$$

Since $t_{\overset{\to }{x}}$ is a constant with respect to the weights, we obtain:

(13) $$\frac{\partial L}{\partial w_{ij\ldots r}}=\sum_{(\overset{\to }{x},t_{\overset{\to }{x}})\in D}\left(t_{\overset{\to }{x}}-u\left(\overset{\to }{x}\right)\right)x_{1}^{i}x_{2}^{j}\cdots x_{k}^{r}\;.$$

This demonstrates that polynomial units are inherently differentiable, making them well-suited for standard and efficient gradient-based optimization techniques.

As a final note, it is well established that polynomials are dense in the space of continuous functions defined over compact domains (Stone–Weierstrass theorem). Consequently, sufficiently high-degree polynomial expansions can approximate continuous decision boundaries under appropriate regularity conditions. In this work, however, we focus on bounded-degree constructions and empirical evaluation, rather than on theoretical approximation guarantees.

### 3.4. Interpretability of Polynomial Perceptrons

The interpretability advantage of PPs lies in the explicit representation of interaction terms, enabling direct inspection of individual and joint feature contributions. A central structural property of a PP is its exact additive decomposition into linear and higher-order interaction components:

(14) $$u\left(\overset{\to }{x}\right)=\underset{\mathrm{linear}\;\mathrm{component}}{\underset{⏟}{w_{0}+\sum_{i=1}^{k}w_{i}x_{i}}}+\underset{\mathrm{interaction}\;\mathrm{component}}{\underset{⏟}{\sum_{\begin{array}{c} |\alpha |\geq 2\\ \alpha \in A \end{array}}w_{\alpha }{\overset{\to }{x}}^{\left(\alpha \right)}}},$$

where $\alpha =(\alpha_{1},\ldots ,\alpha_{k})$ is a multi-index, $\left|\alpha \right|=\sum_{i=1}^{k}\alpha_{i}$ denotes the total degree of the monomial, and A is the set of all multi-indices defining the monomial basis up to degree *d* (as defined in Equation (6)). This decomposition is intrinsic to the model definition and does not rely on post hhoc approximation or surrogate explanation techniques. Each term contributes explicitly and algebraically to the final output of *u*. Formally, the marginal influence of any input variable $x_{j}$ to the output $u(\overset{\to }{x})$, can be expressed as follows:

(15) $$\frac{\partial u(\overset{\to }{x})}{\partial x_{j}}=w_{j}+\sum_{|\alpha |\geq 2}w_{\alpha }\alpha_{j}x_{j}^{\alpha_{j}-1}\prod_{i\neq j}x_{i}^{\alpha_{i}}.$$

The term $w_{j}$ corresponds to the direct linear effect of $x_{j}$. It is constant across the input space and represents a global marginal influence, independent of other variables. On the other hand, each higher-order monomial contributes through the term:

$$w_{\alpha }\alpha_{j}x_{j}^{\alpha_{j}-1}\prod_{i\neq j}x_{i}^{\alpha_{i}},$$

which depends on both $x_{j}$ and the remaining variables. These terms encode interaction effects, meaning that the influence of $x_{j}$ becomes context-dependent. In particular:

- If $\alpha_{j}=0$, the monomial does not contribute to the marginal influence of $x_{j}$.
- If $\alpha_{j}=1$, the interaction term scales proportionally with the product of the other involved variables.
- If $\alpha_{j}\geq 2$, the influence of $x_{j}$ becomes nonlinear even in isolation.

The coefficients $w_{\alpha }$ thus admit a direct behavioral interpretation in terms of local sensitivity, allowing the model’s decision mechanism to be analyzed analytically. Consider a second-order interaction term of the form

(16) $$w_{ij}x_{i}x_{j}.$$

The coefficient $w_{ij}$ quantifies the joint influence of $x_{i}$ and $x_{j}$ on the output. Unlike linear coefficients, which measure isolated marginal effects, interaction coefficients encode synergy or antagonism between variables. The value of $w_{ij}$ can be determined from higher-order derivatives. In particular:

(17) $$\frac{\partial^{2}u\left(\overset{\to }{x}\right)}{\partial x_{i}\partial x_{j}}=w_{ij}.$$

This implies that the interaction coefficient coincides with the mixed second-order partial derivative of the model. Consequently, it provides an exact and analytical measure of the curvature induced by the joint variation in $x_{i}$ and $x_{j}$. More generally, for a higher-order monomial, the associated coefficient corresponds to the respective higher-order mixed derivative of the function.

(18) $$w_{\alpha }{\left(\overset{\to }{x}\right)}^{\alpha },$$

The coefficient satisfies:

(19) $$\frac{\partial^{\left|\alpha \right|}u\left(\overset{\to }{x}\right)}{\partial x_{1}^{\alpha_{1}}\cdots \partial x_{k}^{\alpha_{k}}}=w_{\alpha }.$$

where each coefficient directly corresponds to a pure higher-order interaction effect. This establishes a one-to-one relationship between polynomial coefficients and structured interaction strengths. In summary, the Polynomial Perceptron admits a hierarchical interpretability structure:

- First-order terms represent global linear effects.
- Second-order terms represent pairwise interactions.
- Higher-order terms represent multi-variable cooperative effects.

Interpretability, therefore, emerges as an inherent mathematical property of the PP’s architecture rather than as an approximation-based explanatory add-on. In the subsequent sections, we empirically demonstrate that the PP, particularly its explicit modeling of nonlinear interactions, enables effective and competitive performance across diverse supervised learning tasks.

## 4. Polynomial Perceptrons in Practical Use Cases

To illustrate the practical capabilities of PPs, we present a series of progressively complex use cases that underscore their versatility across a range of supervised learning tasks. Starting from simple binary classification scenarios, we extend the application of PPs to more sophisticated domains, including image classification and natural language processing. This incremental approach not only highlights the expressive power of PPs in capturing nonlinear relationships across diverse data modalities but also emphasizes their inherent interpretability. In each case, we demonstrate how the polynomial structure enables the transparent attribution of predictive outcomes to specific input features, thereby offering meaningful insights into the model’s decision-making process regardless of task complexity.

### 4.1. Binary Classification

Binary classification constitutes a foundational task in supervised learning, wherein the objective is to assign each input instance to one of two distinct categories based on a corresponding set of observed features. Traditional linear models, such as the perceptron, often exhibit limited effectiveness when the underlying class distributions are not linearly separable. In such scenarios, capturing the complexity of the decision boundary requires modeling higher-order interactions among input features. To address this limitation, we introduce the PP as a principled and interpretable approach capable of capturing non-linear relationships within the input space. Below, we detail how the PP architecture enables effective learning in non-linear classification settings through explicit polynomial expansions.

#### 4.1.1. Architecture

**Figure 2:**
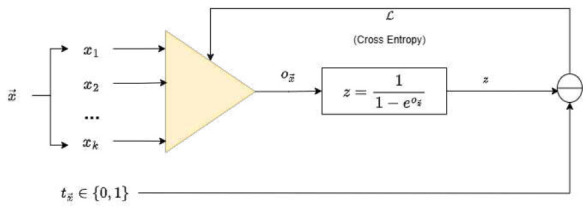
Polynomial perceptron for binary classification.

The proposed PP explicitly constructs nonlinear combinations of input features, effectively expanding the representational capacity of the feature space. This expansion enables the modeling of intricate, nonlinear decision surfaces that cannot be captured by linear transformations alone. To this end, the feature vector $\overset{\to }{x}$ is propagated through the polynomial unit $u(\overset{\to }{x})$, yielding an output value $o_{\overset{\to }{x}}$. The output is subsequently transformed by a sigmoid activation function, producing a transformed value z∈(0,1), which can be interpreted as the estimated probability that $\overset{\to }{x}$ belongs to one of the two target classes. The discrepancy between *z* and the ground truth class $t_{\overset{\to }{x}}\in \left\{0,1\right\}$ can be quantified using the cross-entropy loss function. The loss is minimized via a gradient-based optimization algorithm, updating iteratively the coefficients $w_{ij\ldots r}$ in the direction of the negative gradient. This process is schematically illustrated in Figure 2.

#### 4.1.2. Evaluation

**Figure 3:**
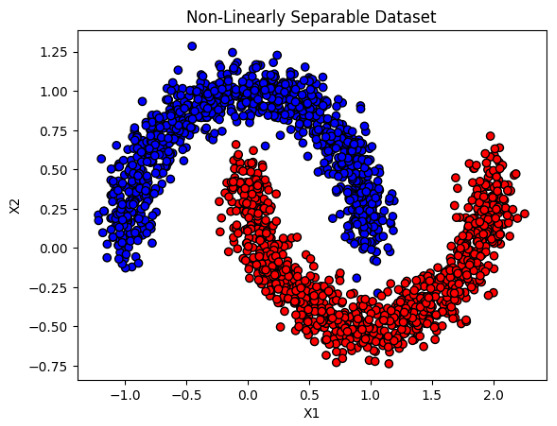
Synthetic non-linearly separable dataset used for evaluating binary classification models. The dataset is generated under controlled conditions to facilitate comparative analysis of model behavior. Each sample belongs to one of two classes, represented by distinct colors.

We assess whether modeling interactions directly in the input space via PPs can achieve predictive performance comparable to that of SVMs with nonlinear kernels and MLPs, which encode nonlinearity through implicit feature mappings or hidden-layer transformations. This comparison allows us to isolate the contribution of explicit interaction terms while maintaining consistent experimental conditions across models. To ensure a controlled and informative comparison, multiple configurations of the PP were evaluated against representative configurations of SVM and MLP, as summarized in Table 1. Specifically, PP models of increasing polynomial degree were considered (degrees d=1,2,3), allowing us to examine the trade-off between expressive capacity and parameter growth. A benchmark dataset, such as the one illustrated in Figure 3, was selected given its complex and non-linear decision boundaries.

Given the relatively simple and controlled structure of the evaluated models, characterized by bounded-degree polynomial expansions in the case of the Polynomial Perceptron (PP) and shallow architectures for baseline models, no dropout or advanced regularization techniques were employed. Instead, model capacity was explicitly regulated through architectural parameters, such as the polynomial degree in PP and the number of hidden units in multilayer perceptrons (MLPs). This design enables a direct and interpretable comparison of expressivity as a function of parameter growth. Standard training-validation splits were employed to monitor generalization performance and mitigate overfitting, ensuring that observed gains are attributable to controlled increases in model capacity rather than regularization effects. To ensure the validity and fairness of the comparison, all models were trained under consistent experimental conditions, incorporating standardized preprocessing procedures, cross-validation protocols, and systematic hyperparameter optimization.

**Figure 4:**
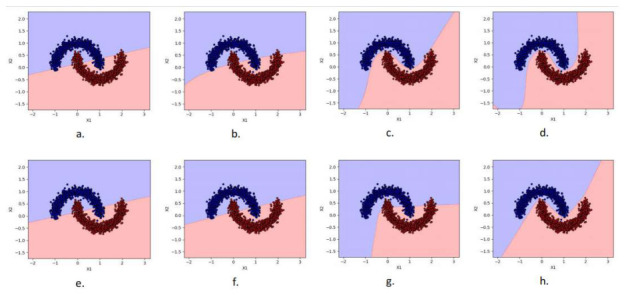
Decision boundaries illustrating the progressive nonlinear expressiveness of the evaluated models on a binary classification task: (**a**) Polynomial Perceptron (PP) with d=1; (**b**) PP with d=2; (**c**) PP with d=3; (**d**) SVM with RBF kernel; (**e**–**h**) MLPs with one hidden layer of 2, 4, 8, and 16 neurons, respectively.

Figure 4 presents the decision boundaries learned by the evaluated models, illustrating their differing abilities to represent nonlinear decision regions. In the visualization, red indicate a positive class whereas blue represent a negative class. The linear PP (d=1) fails to separate the data. Introducing second-order polynomial terms (d=2) produces a curved boundary that closely approximates the intrinsic geometry of the dataset. Higher-degree terms provide limited refinement. SVM and MLP models achieve similar separation through implicit nonlinear transformations. The results in Table 1 quantify the predictive performance and model complexity. As observed, the linear PP (d=1) and quadratic PP (d=2) fail to adequately capture the nonlinear structure of the dataset. However, increasing the degree to d=3 produces a substantial improvement, reaching an accuracy of 0.9967 with only 10 parameters. This performance matches that of an MLP with 16 hidden neurons (65 parameters) and approaches that of the SVM with RBF kernel (0.9983).

Although SVM and small MLPs achieve comparable predictive performance through implicit nonlinear transformations, in these models, the order, strength, and contribution of interactions remain analytically inaccessible. Thus, the contribution of the proposed model is not merely competitive predictive accuracy, but the combination of nonlinear expressiveness and structural interpretability, which is not inherently available in kernel-based or hidden-layer representations.

#### 4.1.3. Interpretability

The contribution of an individual feature can be computed by aggregating: (i) the coefficients of all univariate monomials involving only that feature, and (ii) the coefficients of multivariate monomials in which the feature participates, normalized by the number of variables in each respective interaction term. This procedure leverages the exact additive decomposition of the polynomial model. It distributes interaction effects proportionally among the participating variables, yielding an attribution mechanism that is intrinsic to the model’s parametric structure.

However, a naive aggregation of coefficients may lead to mathematically inconsistent attributions. Simply summing all coefficients associated with a given variable ignores the heterogeneous structure of polynomial terms. In particular, high-order monomials and mixed-order interactions (e.g., $x_{i}^{3}$, $x_{i}^{2}x_{j}$, or $x_{i}x_{j}^{2}$) can disproportionately influence the resulting importance measure if their contributions are not distributed according to their structural participation. Such direct summation implicitly assumes equal attribution of entire coefficients to each participating variable, which overestimates the influence of variables appearing in interaction terms and biases the interpretation toward higher-degree components. To address this issue, we introduce a formally grounded and structurally consistent attribution scheme that allocates each monomial’s contribution proportionally to the exponent with which each variable participates. Consider a binary classifier of the form:

$$f\left(\overset{\to }{x}\right)=g\left(u\left(\overset{\to }{x}\right)\right),$$

where $u(\overset{\to }{x})$ is a PP defined as follows:

$$u\left(\overset{\to }{x}\right)=\sum_{\alpha }w_{\alpha }{\left(\overset{\to }{x}\right)}^{\alpha },\;{\left(\overset{\to }{x}\right)}^{\alpha }=\prod_{k=1}^{d}x_{k}^{\alpha_{k}},$$

with multi-index $\alpha \in N^{d}$ and total degree $\left|\alpha \right|=\sum_{k=1}^{d}\alpha_{k}$. The function g:R→R is an arbitrary activation function. We define the global structural contribution of variable $x_{i}$ at the polynomial level as follows:

$$C_{i}=\sum_{\alpha :\alpha_{i}>0}\frac{\alpha_{i}}{\left|\alpha \right|}\;w_{\alpha }.$$

**Theorem** **1** (Exponent-Proportional Structural Attribution). *Let $f\left(\overset{\to }{x}\right)=g\left(u\left(\overset{\to }{x}\right)\right)$ with $u(\overset{\to }{x})$ polynomial. Then the attribution measure $\left\{C_{i}\right\}$ satisfies:*

*1*. *(Structural Conservation)*
$$\sum_{i=1}^{d}C_{i}=\sum_{\alpha }w_{\alpha }.$$
*2*. *(Activation Independence) The quantities $\left\{C_{i}\right\}$ depend exclusively on the polynomial structure of $u(\overset{\to }{x})$ and are independent of the choice of activation function g.*
*3*. *(Monotonic Invariance) If g is strictly monotonic, then the relative influence ordering induced by $\left\{C_{i}\right\}$ is preserved in $f(\overset{\to }{x})$.*

**Proof.**  (1) Structural Conservation.

For any monomial with |α|>0:

$$\sum_{i=1}^{d}\frac{\alpha_{i}}{\left|\alpha \right|}=\frac{1}{\left|\alpha \right|}\sum_{i=1}^{d}\alpha_{i}=1.$$

Therefore:

$$\sum_{i=1}^{d}C_{i}=\sum_{\alpha }\left(\sum_{i=1}^{d}\frac{\alpha_{i}}{\left|\alpha \right|}\right)w_{\alpha }=\sum_{\alpha }w_{\alpha }.$$

(2) Activation Independence.

The definition of $C_{i}$ depends solely on the coefficients $\left\{w_{\alpha }\right\}$ of the polynomial $u(\overset{\to }{x})$. Since *g* operates only on the scalar output of *u*, the decomposition of structural contributions remains unaffected by the functional form of *g*.

(3) Monotonic Invariance.

If *g* is strictly monotonic, then for any ${\overset{\to }{x}}_{1},{\overset{\to }{x}}_{2}$:

$$u\left({\overset{\to }{x}}_{1}\right)>u\left({\overset{\to }{x}}_{2}\right)\;⟺\;g\left(u\left({\overset{\to }{x}}_{1}\right)\right)>g\left(u\left({\overset{\to }{x}}_{2}\right)\right).$$

Hence, structural differences induced by $\left\{C_{i}\right\}$ at the logit level are preserved after activation. □

Since $u(\overset{\to }{x})$ represents the logit, the signed evidence separating the two classes, a positive $C_{i}$ indicates that, in aggregate, $x_{i}$ contributes positively to the logit and therefore increases evidence toward the positive class. Conversely, a negative $C_{i}$ indicates that $x_{i}$ contributes negatively to the logit, reinforcing evidence toward the negative class. Thus, $C_{i}$ does not represent a contribution to a specific class independently, but rather a contribution to the signed decision margin that discriminates between the two classes.

**Figure 5:**
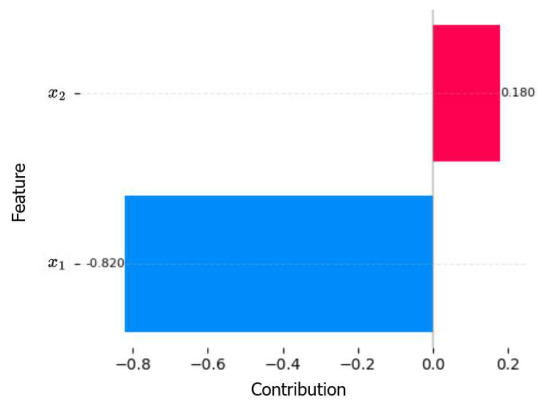
Illustration of feature-wise contribution analysis for a test instance using a PP. Each bar represents the individual contribution of a feature $x_{i}$ to the model’s output, highlighting both positive and negative influences. Notably, feature $x_{2}$ contributes positively (0.180), while feature $x_{1}$ has a negative impact (−0.820), offering interpretable insight into the prediction mechanism.

This strategy enables a direct and analytically grounded quantification of the marginal contribution of each feature $x_{i}\in \overset{\to }{x}$ to the model’s prediction. For instance, in Figure 5, the feature $x_{2}$ contributes positively with a value of 0.180, while $x_{1}$ contributes negatively with −0.820. In the visualization, red indicate a positive contribution whereas blue represent a negative contribution. Note that attributions are not derived from surrogate approximations, sampling procedures, or local linearizations. Rather, they follow deterministically from the learned polynomial coefficients themselves. Unlike post hhoc explanation frameworks such as SHAP or LIME, which approximate feature influence after training and depend on perturbation strategies or auxiliary models, the proposed approach derives interpretability directly from the functional form of the predictor.

### 4.2. Multiclass Classification

Multiclass classification is a generalization of the binary classification problem, where the objective is to assign each input instance to one of three or more mutually exclusive categories. Here, we describe the architecture for addressing this problem using PPs, detailing the evaluation methodology to assess its performance and offering a comprehensive discussion on the interpretability of the resulting model.

#### 4.2.1. Architecture

**Figure 6:**
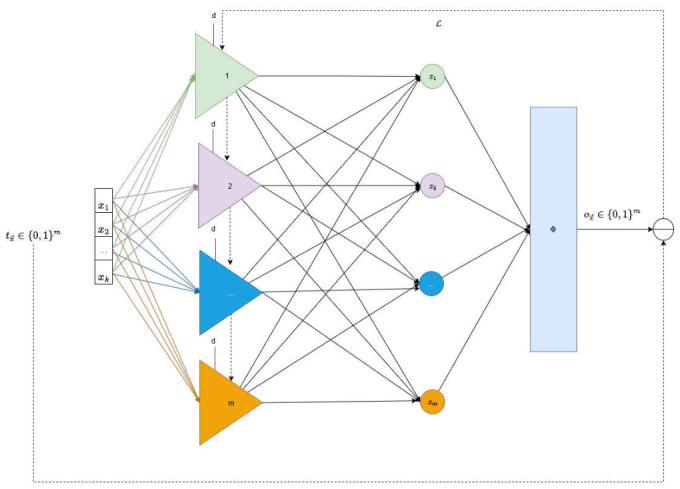
Neural network with polynomial units for multiclass classification.

To extend the PP’s capabilities to a multiclass classification task, we propose an architectural design representing each class by a dedicated, class-specific polynomial unit $u_{i}\left(\overset{\to }{x}\right)$. Each unit computes a raw activation score $z_{i}$, reflecting the degree of alignment between the input vector $\overset{\to }{x}$ and the *i*-th class prototype. These raw scores are then aggregated and normalized using a function Φ (typically a softmax function), producing a probability distribution over possible classes, wherein the predicted label corresponds to the class with the highest probability. This architecture is schematically depicted in Figure 6. During training, the predicted class label vector $o_{\overset{\to }{x}}\in {\left\{0,1\right\}}^{m}$ is compared to the ground truth $t_{\overset{\to }{x}}\in {\left\{0,1\right\}}^{m}$, where m≥3 denotes the number of classes. A suitable loss function, such as cross-entropy, is employed to quantify the discrepancy between the predicted and actual labels. Through gradient-based optimization methods, this error signal then updates the polynomial coefficients in all units $u(\overset{\to }{x})$.

#### 4.2.2. Evaluation

**Figure 7:**
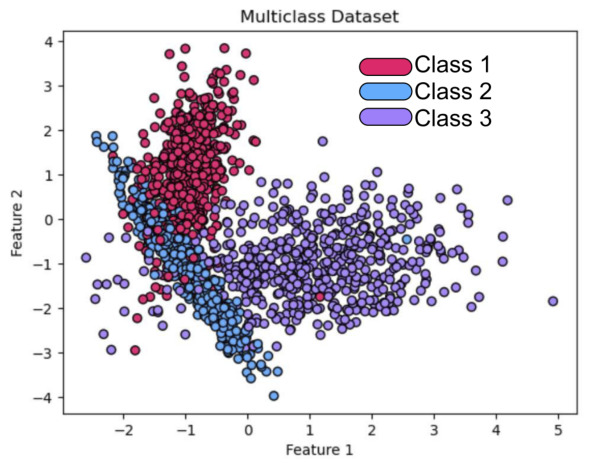
Synthetic non-linearly separable dataset used for evaluating multiclass classification models. The data are generated under controlled conditions to enable systematic comparison of model behavior. Each sample is assigned to one of three classes, represented by distinct colors.

To evaluate the effectiveness of the proposed multiclass PP model, we conducted experiments using a synthetically generated dataset specifically designed to introduce class overlap. The dataset consists of multiple classes whose instances are sampled from multivariate distributions with partially intersecting support regions in the input space, as depicted in Figure 7. This configuration imposes a challenging classification scenario that simulates real-world conditions where class boundaries are not linearly separable. Each data point $\overset{\to }{x}\in R^{k}$, with k=2, was assigned a ground truth label $t_{\overset{\to }{x}}$.

**Figure 8:**
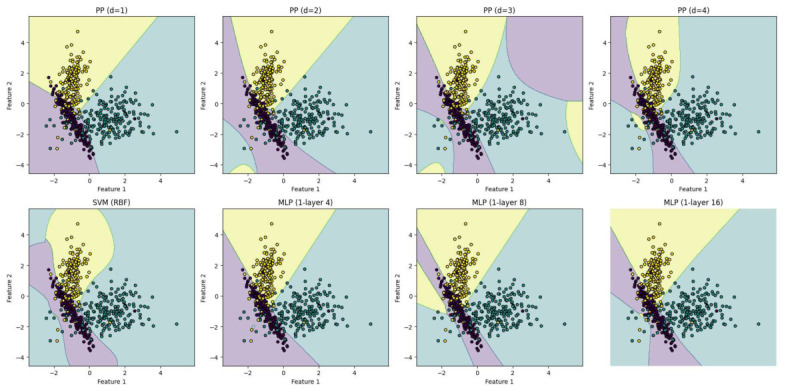
Decision boundaries produced by each model on the multiclass dataset. The visualization highlights how each model partitions the input space and reveals differences in generalization capacity and boundary complexity. Notably, the PP model achieves smooth yet expressive boundaries, illustrating its ability to capture nonlinear patterns with low model complexity.

We conducted a comprehensive ablation analysis to evaluate the proposed PP model under varying polynomial degrees and to compare its performance against widely used nonlinear classification baselines, namely SVM with RBF kernel and shallow MLP architectures. All models were trained and evaluated using identical data splits and optimization protocols to ensure a controlled and fair comparison. Performance was assessed primarily in terms of classification accuracy on both training and test sets, complemented by confusion matrix analysis to examine class-wise behavior. This ablation framework allows us to systematically analyze the impact of increasing polynomial degree in the PP model and to contrast explicit polynomial interaction modeling against implicit nonlinear representations learned by kernel methods and neural networks, as illustrated in Figure 8.

Table 2 presents the multiclass ablation results. A clear monotonic performance improvement is observed as the polynomial degree increases from d=1 to d=4, confirming that higher-order interactions progressively enhance the model’s ability to capture nonlinear class boundaries. The transition from linear (d=1) to quadratic and cubic terms yields the most substantial gains, indicating that explicit interaction modeling is essential for this dataset. Importantly, the PP with d=3 achieves the same accuracy as the SVM with RBF kernel (0.8667), while the PP with d=4 slightly surpasses it (0.8700). This demonstrates that explicit polynomial expansions can match the representational capacity of kernel-based methods without relying on implicit high-dimensional mappings. When compared to shallow MLPs, the PP exhibits competitive behavior across all configurations. The MLP with 8 neurons attains the highest accuracy (0.8733), but requires 51 parameters, whereas the PP with d=4 achieves nearly identical performance (0.8700) using 45 explicitly interpretable coefficients. Notably, increasing the MLP size to 16 neurons does not yield further improvement, suggesting diminishing returns from hidden-layer scaling. These results highlight two central findings: (i) performance gains in the PP are directly attributable to the controlled increase in polynomial degree, as shown by the ablation analysis, and (ii) explicit polynomial interaction modeling provides competitive accuracy relative to kernel and neural approaches while preserving structural interpretability. Unlike SVMs, whose effective parameters depend on support vectors and remain implicit in the dual representation, the PP maintains a fixed and directly interpretable parametric form, where each coefficient corresponds to a specific monomial term.

The above findings underscore the strength of the PP architecture as a lightweight yet effective alternative for multiclass classification. Its ability to capture nonlinear relationships through polynomial interactions (as illustrated in Figure 8), while maintaining a minimal footprint, highlights its potential for deployment in environments where model size, interpretability, and efficiency are critical. These results also indicate that the multiclass extension retains the core advantages previously observed: a reduced number of trainable parameters compared to other architectures and a high level of interpretability. Specifically, each polynomial unit can be directly examined to understand the contribution of input features to the classification decision.

#### 4.2.3. Interpretability

An important advantage of the PP multiclass architecture lies in its inherent interpretability.

Unlike deeper neural architectures, which may involve multiple layers of nonlinear transformations, both the Polynomial Perceptron and a single-layer MLP can be interpreted as linear models over their respective feature representations. In this sense, interpretability does not arise from the classifier itself, but from the structure of the input features.

In the case of the proposed model, the explicit polynomial expansion provides a direct correspondence between learned coefficients and specific monomial interactions among input variables. This allows individual terms (e.g., pairwise or higher-order interactions) to be examined explicitly. By contrast, a standard MLP operating on the original input features does not explicitly encode such interactions unless they are introduced through feature engineering or deeper architectures.

Therefore, the interpretability of the proposed formulation lies in the transparency of its feature construction rather than in the linear decision function per se.

For instance, the polynomial basis, with d=3, for each class includes the following monomial terms:

$$\{1,\;x_{1},\;x_{2},\;x_{1}^{2},\;x_{1}x_{2},\;x_{2}^{2},\;x_{1}^{3},\;x_{1}^{2}x_{2},\;x_{1}x_{2}^{2},\;x_{2}^{3}\}\;.$$

This formulation corresponds to a complete third-degree polynomial in two variables, with 10 terms per class. Each polynomial model can be systematically analyzed to quantify the contribution of individual features to the corresponding class prediction, following a methodology analogous to that employed in the binary classification setting. This approach enables an interpretable decomposition of the decision function, whereby the influence of each feature is explicitly derived from the structure and coefficients of the polynomial. In particular, for the synthetic dataset under consideration, the resulting polynomials of the classification model, for d=3, are as follows:

$$\begin{array}{cc} \mathrm{Class}\;1: & \\ \; & -0.0478-1.8661\;x_{1}+1.7997\;x_{2}-0.2358\;x_{1}^{2}-0.2482\;x_{1}x_{2}+0.3028\;x_{2}^{2}\\ \; & +0.4872\;x_{1}^{3}-0.0571\;x_{1}^{2}x_{2}-0.9416\;x_{1}x_{2}^{2}+0.1946\;x_{2}^{3}\\ \mathrm{Class}\;2: & \\ \; & 0.3225-1.5243\;x_{1}-1.3836\;x_{2}+2.3139\;x_{1}^{2}+1.2600\;x_{1}x_{2}+0.3697\;x_{2}^{2}\\ \; & -1.3172\;x_{1}^{3}-0.8958\;x_{1}^{2}x_{2}-1.3943\;x_{1}x_{2}^{2}-0.8025\;x_{2}^{3}\\ \mathrm{Class}\;3: & \\ \; & -0.2735+2.5173\;x_{1}-0.8054\;x_{2}+0.3645\;x_{1}^{2}+0.6265\;x_{1}x_{2}-0.8637\;x_{2}^{2}\\ \; & -0.6833\;x_{1}^{3}-1.5852\;x_{1}^{2}x_{2}+0.0140\;x_{1}x_{2}^{2}+0.1144\;x_{2}^{3} \end{array}$$

Formally, the multiclass version of PPs with degree d=3 defines, for each class c∈{1,2,3}, a cubic decision function:

$$u_{c}\left(\overset{\to }{x}\right)=\sum_{|\alpha |\leq 3}w_{\alpha }^{\left(c\right)}{\left(\overset{\to }{x}\right)}^{\alpha },$$

where $\overset{\to }{x}=\left(x_{1},x_{2}\right)$ and ${\left(\overset{\to }{x}\right)}^{\alpha }=x_{1}^{\alpha_{1}}x_{2}^{\alpha_{2}}$. The coefficients $w_{\alpha }^{\left(c\right)}$ quantify the contribution of each monomial term to the class-specific logit $u_{c}\left(\overset{\to }{x}\right)$. The linear coefficients determine the first-order sensitivity of each class score with respect to the input variables. For instance, in Class 1:

$$-1.8661\;x_{1}+1.7997\;x_{2},$$

indicates that increasing $x_{1}$ decreases the evidence for Class 1, while increasing $x_{2}$ strengthens it. Formally:

$$\frac{\partial u_{1}}{\partial x_{1}}=-1.8661+O\left(x\right),\;\frac{\partial u_{1}}{\partial x_{2}}=1.7997+O\left(x\right),$$

where $O(\overset{\to }{x})$ denotes higher-order corrections from nonlinear terms. Analogously, Class 3 exhibits a strong positive linear dependence on $x_{1}$ (+2.5173) and a negative dependence on $x_{2}$ (−0.8054), indicating that $x_{1}$ primarily drives separation toward Class 3 in regions where nonlinear effects are moderate. These linear coefficients, therefore, define the dominant global orientation of the decision boundaries. Second-order monomials introduce curvature and pairwise feature interactions. For example, in Class 2:

$$2.3139\;x_{1}^{2}+1.2600\;x_{1}x_{2}+0.3697\;x_{2}^{2},$$

the large positive coefficient of $x_{1}^{2}$ implies that extreme values (positive or negative) of $x_{1}$ increase the evidence for Class 2. The mixed term $1.2600\;x_{1}x_{2}$ captures interaction effects: the contribution depends on the joint sign and magnitude of both variables, effectively rotating and bending the decision boundary. In contrast, Class 3 contains a strong negative quadratic effect in $x_{2}^{2}$ (−0.8637), meaning that large magnitudes of $x_{2}$ suppress evidence for that class. These quadratic coefficients, therefore, shape the curvature of the separating surfaces and allow the model to adapt to overlapping class regions. Finally, third-order terms refine local geometric behavior and enable asymmetric distortions of the decision function. For instance, in Class 1:

$$0.4872\;x_{1}^{3}-0.9416\;x_{1}x_{2}^{2},$$

The cubic monomials introduce directional nonlinear amplification. The term $x_{1}^{3}$ produces asymmetric growth depending on the sign of $x_{1}$, while $x_{1}x_{2}^{2}$ couples a linear dependence on $x_{1}$ with quadratic magnitude in $x_{2}$, enabling complex bending of the decision boundary in specific regions of the input space. Similarly, the large negative cubic coefficients in Class 2 ($-1.3172\;x_{1}^{3}$, $-1.3943\;x_{1}x_{2}^{2}$) indicate strong nonlinear suppression in certain directions, which compensates for the positive quadratic expansion and stabilizes class-specific regions.

Crucially, each coefficient corresponds to a specific and identifiable geometric effect: linear terms control orientation, quadratic terms control curvature and pairwise interaction strength, and cubic terms modulate asymmetric nonlinear distortions. Because the decision functions are explicit polynomials, these contributions are analytically tractable through partial derivatives and direct coefficient inspection. The model’s behavior is therefore structurally interpretable: class evidence arises from a transparent superposition of well-defined monomial effects rather than from distributed hidden activations or implicit kernel embeddings.

To obtain a global measure of the contribution of each variable, in accordance with the proposed architecture (see Section 4.2.1), we consider a multiclass classifier with *m* classes as a function:

$$f\left(\overset{\to }{x}\right)=g\left(u_{1}\left(\overset{\to }{x}\right),\ldots ,u_{m}\left(\overset{\to }{x}\right)\right),$$

where $u_{c}\left(\overset{\to }{x}\right)$ is a class-specific polynomial perceptron defined as follows:

$$u_{c}\left(\overset{\to }{x}\right)=\sum_{\alpha }w_{\alpha }^{\left(c\right)}{\left(\overset{\to }{x}\right)}^{\alpha },\;c=1,\ldots ,m,$$

and $g:R^{m}\to R^{m}$ is an arbitrary activation function (e.g., softmax, normalized exponential, identity, etc.). For each class *c*, we define the structural contribution of variable $x_{i}$ as follows:

$$C_{i}^{\left(c\right)}=\sum_{\alpha :\alpha_{i}>0}\frac{\alpha_{i}}{\left|\alpha \right|}\;w_{\alpha }^{\left(c\right)}.$$

**Figure 9:**
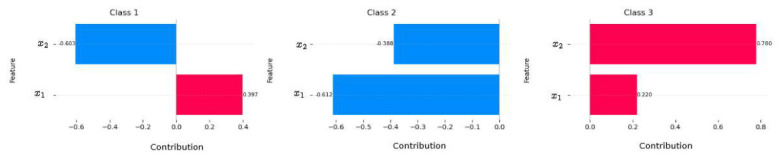
Class-specific structural feature contributions $C_{i}^{\left(c\right)}$ derived from the induced polynomial logits. For each class *c*, the values quantify the intrinsic contribution of each feature $x_{i}$ to the corresponding logit $u_{c}\left(x\right)$, computed directly from the polynomial coefficients. Positive values indicate structural support for the class, whereas negative values indicate structural opposition. The relative magnitudes reflect how the model internally allocates feature importance across classes, independently of the output activation function.

This quantity measures the global structural influence of $x_{i}$ on the class-*c* decision logit $u_{c}\left(\overset{\to }{x}\right)$. Figure 9 illustrates the contributions for the two-dimensional input space induced by the dataset. For Class 1, the value $C_{2}^{\left(1\right)}=-0.603$ indicates that $x_{2}$ exerts a net negative structural influence on the corresponding logit $u_{1}\left(\overset{\to }{x}\right)$, while $C_{1}^{\left(1\right)}=0.397$ shows that $x_{1}$ contributes positively. In other words, increases in $x_{1}$ systematically strengthen the structural support for Class 1, whereas increases in $x_{2}$ weaken it. For Class 2, both features exhibit negative contributions, i.e., $C_{1}^{\left(2\right)}<0$ and $C_{2}^{\left(2\right)}<0$. This implies that, at the structural level of the logit, the learned polynomial assigns both variables a diminishing effect on the evidence for this class. The model, therefore, encodes Class 2 as being structurally disfavored by increases in either coordinate. In contrast, for Class 3, both contributions are positive, with $C_{1}^{\left(3\right)}=0.220$ and $C_{2}^{\left(3\right)}=0.780$. The larger magnitude of $C_{2}^{\left(3\right)}$ indicates that $x_{2}$ plays the dominant structural role in increasing the logit of Class 3. This asymmetry reveals how the model internally allocates importance across features for different classes.

Importantly, these quantities are not obtained via gradient-based sensitivity analysis, sampling approximations, or surrogate explanation models. They arise directly from the algebraic structure of the classifier and therefore reflect intrinsic properties of the learned decision function. Because the contributions are defined at the logit level, they remain independent of the choice of output activation (e.g., softmax) and thus provide a stable, activation-agnostic measure of feature influence.

This structural perspective offers a transparent alternative to post hhoc interpretability methods: rather than explaining predictions after the fact, it exposes how the model’s polynomial representation encodes class-specific evidence directly in its parameters.

### 4.3. Image Classification

To extend the PP model to more complex tasks, we explore its applicability within the context of image classification, a domain traditionally addressed using deep learning techniques. This extension entails integrating the feature extraction process directly into the learning pipeline, aligning with the core principle of representation learning in deep architectures.

#### 4.3.1. Preliminary Architecture

**Figure 10:**
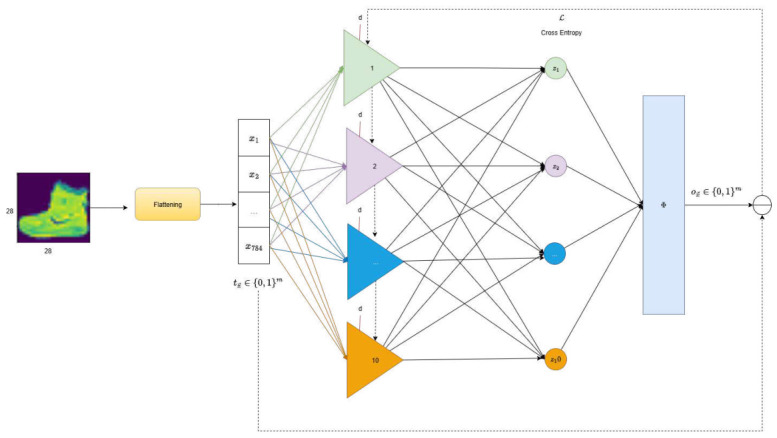
Preliminary architecture for extending the PP model to image classification tasks. In this design, input images are flattened into one-dimensional vectors before being processed by class-specific polynomial units. While this approach enables compatibility with the PP framework, it disregards spatial relationships within the image and leads to a combinatorial increase in the number of polynomial terms, thus limiting scalability and representational efficiency.

As a preliminary approach, we employed a straightforward flattening procedure to transform image data into a vectorized format compatible with the input requirements of the polynomial units in the PP model, as depicted in Figure 10. For ilustration proposes, we consider a classification task involving single-channel images of size 28 × 28 pixels, corresponding to 10 different classes. Each image undergoes a simple flattening process—used as a basic feature extraction method—to produce an input vector $\overset{\to }{x}\in R^{784}$, which is then processed by a neural network built from polynomial units.

While this strategy enables the direct application of the PP framework to image classification tasks, it exhibits two critical limitations. First, the flattening process inherently disrupts the spatial structure of the image, eliminating local correlations that are essential for capturing meaningful visual patterns. Second, this approach leads to a combinatorial explosion in the number of polynomial terms, particularly as input dimensionality increases.

Flattening a 28 × 28 image yields a 784-dimensional input vector. For a PP of degree d=2, the number of monomial terms in a complete expansion is given by k+22=308,505 terms. Although this count remains finite and explicitly parameterized, it illustrates the rapid combinatorial growth of complete polynomial expansions with increasing input dimensionality. Such growth can impose substantial memory and computational demands, motivating the incorporation of more structured and spatially-aware feature extraction mechanisms within the PP architecture.

#### 4.3.2. Robust Architecture

**Figure 11:**
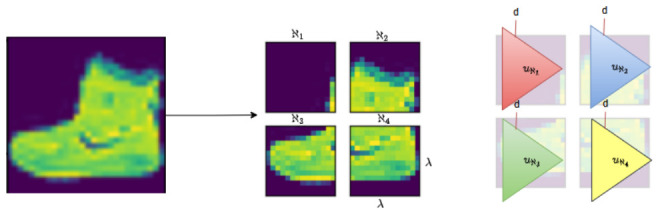
Local feature extraction using polynomial units applied to patches of the input image. The image of size τ×τ is partitioned into sub-images of size λ×λ, and each sub-image is processed by a dedicated polynomial unit.

To address the inherent limitations of the preliminary architecture, we developed a more sophisticated approach incorporating spatially-aware feature extraction mechanisms before the polynomial classification stage. This revised design preserves the geometric relationships among pixels, enabling the PP model to exploit localized and hierarchical patterns within the image. By embedding the feature extraction process into the learning architecture. We propose a localized feature extraction strategy tailored to the structure of the PP model. Rather than flattening the entire image into a single high-dimensional vector, the input image of size τ×τ is partitioned into smaller, non-overlapping patches of size λ×λ, with λ<τ. As depicted in Figure 11, polynomial units are then applied independently to each patch, enabling the model to focus on localized regions of the image.

**Figure 12:**
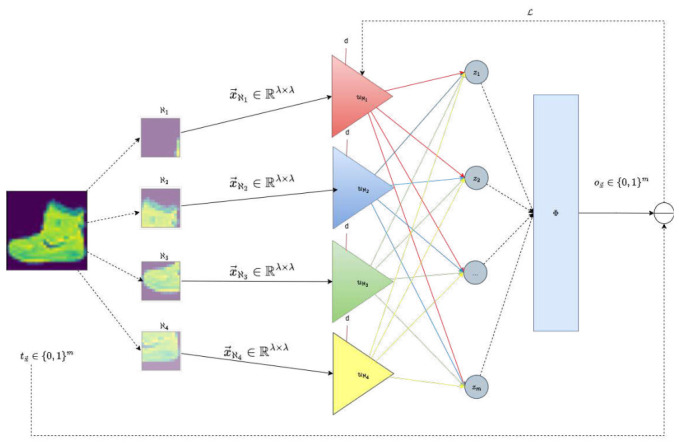
Network architecture using localized polynomial units. Each sub-image ${ℵ}_{i}$ is flattened and processed by a corresponding polynomial unit $u_{{ℵ}_{i}}$, which is fully connected to a shared output layer, enabling the integration of localized representations into a unified classification output. This design preserves spatial locality while maintaining computational efficiency.

This approach offers several key advantages: it preserves spatial coherence, reduces the input dimensionality per unit, and significantly limits the number of polynomial terms required for each local representation. Consequently, it maintains computational tractability while enhancing the model’s capacity to capture local patterns and textures, which are critical for image classification tasks. In this design, each sub-image extracted from the original input and denoted as ${ℵ}_{i}$, is individually flattened into a vector ${\overset{\to }{x}}_{ℵi}$, which serves as the input for a dedicated polynomial unit $u_{ℵi}$. These units operate independently, enabling localized nonlinear transformations that capture region-specific patterns. Each $u_{ℵi}$ is then fully connected to a shared output layer, facilitating the integration of localized representations into a global classification decision. This architecture, illustrated in Figure 12, balances model expressiveness and computational efficiency, while preserving the spatial coherence essential for effective image analysis.

**Figure 13:**
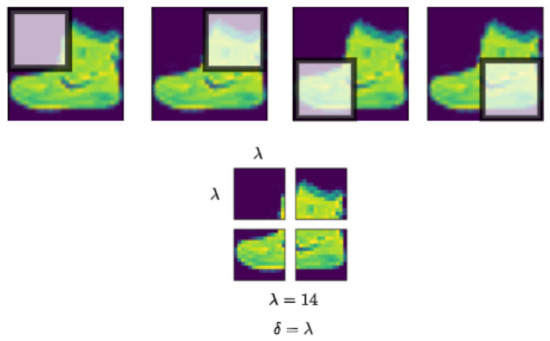
Non-overlapping sub-images generated via a sliding window with offset δ=λ.

Up to this point, the sub-images used in our architecture can be interpreted as resulting from a sliding window process with an offset δ=λ, where each sub-image corresponds to a non-overlapping region of the original image, as illustrated in Figure 13.

**Figure 14:**
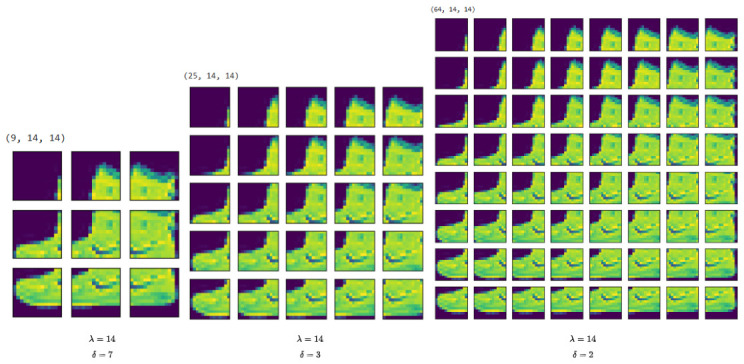
Overlapping sub-images with offsets δ<λ, preserving spatial continuity and fine detail.

Since the sub-images in such initial partitioning scheme are non-overlapping, important spatial context across adjacent regions may be lost. To mitigate this limitation, we propose the use of a smaller stride or offset δ<λ. This results in overlapping sub-images, allowing for shared information between neighboring regions. As illustrated in Figure 14, this strategy enhances spatial continuity and enables the model to capture finer-grained local structures, thereby improving the representation of contextual dependencies within the image.

In this strategy, the number of overlapping pixels between two adjacent sub-images is given by λ−δ, where λ denotes the patch size and δ is the offset or stride. In the case of a single-channel 28 × 28 image, setting λ=14 and using offset values of λ=7, λ=3, and λ=2 results in overlapping regions of 7, 11, and 12 pixels, respectively. The use of overlapping patches plays a critical role in preserving important spatial information, such as edges, corners, and textures—features that are essential for accurate object recognition and structural understanding.

To get an idea of the growth in the number of parameters, let’s look at three architecture examples assuming a single-channel 28 × 28 image.

- **PP-Flat**: Global degree-2 polynomial applied to flattened input.
- **PP-Local (δ=14)**: Non-overlapping localized polynomial units.
- **PP-Local (δ=7)**: Overlapping localized polynomial units.

**P-Flat**: for $\overset{\to }{x}\in R^{784}$ and d=2, this architecture contains:

$$1+784+\frac{784\times 785}{2}=1+784+307,720=308,505$$

terms per output logit. For 10 classes: 308,505×10=3,085,050 parameters.

**PP-Local** (Non-Overlapping Patches): we partition the image into patches of size λ=14, yielding:

$$\frac{28}{14}\times \frac{28}{14}=2\times 2=4\;\mathrm{patches}.$$

Each patch contains:

14×14=196inputs.

A degree-2 polynomial on 196 variables contains:

$$1+196+\frac{196\times 197}{2}=1+196+19,306=19,503$$

parameters. Each patch produces a single scalar output. Total local polynomial parameters:

4×19,503=78,012.

These 4 scalars are connected to a shared 10-class linear layer:

4×10+10=50

additional parameters. Total: 78,012+50=78,062 parameters.

**PP-Local** (Overlapping Patches): we use a stride defined by δ=7, using an offset value of λ=14 which produces:

$$\left\lfloor \frac{\lambda }{\delta }\right\rfloor +1=3$$

patches per dimension, yielding:

3×3=9patches.

Using PPs with d=2, the total local polynomial parameters is:

9×19,503=175,527.

Final linear layer:

9×10+10=100.

Total: 175,527+100=175,627 parameters.

#### 4.3.3. Evaluation

We evaluate the proposed architecture on a benchmark dataset of single-channel 28×28 grayscale images with 10 classes (Fashion-MNIST). All polynomial units were restricted to degree 2 to maintain computational tractability.

As reference points, we implemented lightweight comparison architectures following the same training and evaluation protocol. Rather than introducing significantly smaller convolutional models, we focus on lightweight architectures whose effective capacity remains within a comparable range, enabling a more meaningful comparison across structurally different model families. These comparisons are intended to illustrate relative behavior within this specific setting, without implying structural or universal equivalence across model families. Compared architectures were the following:

- **PP-Flat**: Global degree-2 polynomial applied to flattened input.
- **PP-Local**: Global degree-2 (δ=14) Non-overlapping localized polynomial units.
- **PP-Local**: Global degree-2 (δ=7) Overlapping localized polynomial units.
- **MLP-100**: One hidden layer with 100 units (capacity-matched to PP-Local δ=14).
- **MLP-224**: One hidden layer with 224 units (capacity-matched to PP-Local δ=7).
- **CNN-Low**: Two-layer CNN.
- **CNN-Medium**: Two-layer CNN.

All architectures processed the same 28×28 grayscale inputs across 10 categories. Unless otherwise specified, models were trained using consistent optimization settings so that observed performance differences primarily reflect architectural characteristics within the evaluated configuration rather than differences in training procedures.

For contextual comparison, we implemented MLP architectures whose number of learned scalar coefficients falls within a similar scale to the local PP configurations. For an MLP with one hidden layer of size *h*:

Parameters(h)=784h+h+10h+10=795h+10

To ensure fair comparisons, we match MLP capacity to the number of trainable parameters of each PP configuration, as follows:

- **MLP-100**:
  Parameters(100)=795(100)+10=79,510.

This closely matches PP-Local (δ=14).

- **MLP-224**:
  Parameters(224)=795(224)+10=178,090.

These models were selected to operate within a comparable magnitude of learned coefficients, allowing performance to be examined under approximately capacity-aligned conditions, without implying strict architectural equivalence.

Similarly, we implemented two lightweight CNN baselines following standard two-layer convolutional designs with ReLU activations and max-pooling. Due to structural differences such as weight sharing and local connectivity, exact equality in learned scalar coefficients across architectural families is not attainable. The selected configurations were chosen to fall within a similar scale of learned coefficients relative to the PP-Local variants.

- **CNN-Low**:
  – Conv(1, 32, 3×3) + ReLU.
  – MaxPool(2×2).
  – Conv(32, 64, 3×3) + ReLU.
  – MaxPool(2×2).
  – Fully Connected (to 10 classes).

Total parameters:

$$\left(3^{2}\times 1\times 32+32\right)+\left(3^{2}\times 32\times 64+64\right)+\left(3136\times 10+10\right)=50,186.$$

- **CNN-Medium**:
  – Conv(1, 48, 3×3) + ReLU.
  – Conv(48, 96, 3×3) + ReLU.
  – MaxPool(2×2).
  – Fully Connected (to 10 classes).

Total parameters:

$$\left(3^{2}\times 1\times 48+48\right)+\left(3^{2}\times 48\times 96+96\right)+\left(18,816\times 10+10\right)=230,218.$$

Zero-padding was applied to preserve spatial dimensions before pooling. The number of output channels was adjusted to maintain comparable coefficient scales across models. All experiments were conducted under identical hyperparameter tuning budgets and repeated across N independent random seeds. The resulting test-set accuracies are reported in Table 3. These results should be interpreted as a controlled case study on a single-channel benchmark dataset, rather than as a comprehensive evaluation across image domains.

Within this controlled configuration, local Polynomial Perceptron variants achieve higher accuracy than the evaluated dense MLP baselines operating at a similar scale of learned coefficients. Convolutional networks remain competitive, reflecting the strong inductive bias introduced by weight sharing and spatial locality. In particular, PP-Local (δ=7) attains performance comparable to the CNN-Medium configuration within this dataset setting, while retaining an explicit polynomial representation that allows for direct coefficient inspection. By contrast, the fully global polynomial model exhibits substantial combinatorial growth in learned coefficients without proportional gains in accuracy, suggesting that structured local interaction modeling plays an important role in controlling representational complexity. Overall, these findings illustrate the feasibility of localized polynomial constructions under bounded degree on a single-channel image benchmark. Broader validation on multi-channel datasets and additional image benchmarks remains future work.

#### 4.3.4. Interpretability via Structured Contribution Decomposition

The predictive behavior of architectures such as MLPs and CNNs emerges from cascades of nonlinear transformations across multiple layers. Although highly expressive, these models encode feature influence in a distributed and entangled manner: the contribution of any individual input variable or spatial region is implicitly mediated through layer compositions, activation nonlinearities, and weight sharing. As a result, tracing or quantifying the influence of specific features on the final decision requires post hhoc approximation methods (e.g., gradients or perturbations), rather than direct structural analysis. In contrast, the Polynomial Perceptron (PP) architecture admits an explicit functional representation of the decision rule for each class *c*, expressed as follows:

$$u^{\left(c\right)}\left(\overset{\to }{x}\right)=\sum_{\alpha }w_{\alpha }^{\left(c\right)}\prod_{i}x_{i}^{\alpha_{i}},$$

whose coefficients $w_{\alpha }^{\left(c\right)}$ directly encode the strength of individual features and their interactions. This explicit form enables a principled and transparent decomposition of the prediction into contributions from individual variables or variable subsets. When the image is flattened into a vector representation, the contribution of each input variable can be computed analogously to the multiclass formulation, by proportionally distributing each monomial coefficient according to its exponent share. Formally, the contribution of pixel $x_{i}$ to class *c* is:

$$C_{i}^{\left(c\right)}=\sum_{\alpha :\alpha_{i}>0}w_{\alpha }^{\left(c\right)}\frac{\alpha_{i}}{\left|\alpha \right|}.$$

This provides a global, coefficient-level attribution consistent with the polynomial structure. However, flattening removes the explicit spatial organization of pixels, making it less transparent to identify localized interaction patterns or spatially coherent structures among neighboring pixels.

When applied to image classification with localized polynomial units, interpretability becomes spatially structured. Each polynomial unit operates on a specific image patch $P_{i}$, producing an intermediate scalar output $z_{i}$ (see Figure 12). These local outputs are then linearly combined in the final classification layer. For a model with *m* classes, let $w_{ij}$ denote the weight connecting patch output $z_{i}$ to the logit corresponding to class *j*. The weight $w_{ij}$ directly quantifies the influence of the *i*-th spatial region on the prediction of class *j*. Consequently, the contribution of patch $P_{i}$ to class *j* can be expressed as follows:

$$C_{P_{i}}^{\left(j\right)}=w_{ij}\;z_{i}.$$

This decomposition yields a transparent regional explanation: each class decision is expressed as an explicit weighted sum of localized polynomial responses. In configurations with overlapping patches, multiple polynomial units may depend on shared input pixels. To preserve interpretability and avoid redundant attribution, the contribution assigned to shared regions is averaged across all polynomial units containing those pixels. Formally, if a pixel (u,v) belongs to a set of patches I(u,v), its class-specific contribution is defined as follows:

$$C_{u,v}^{\left(j\right)}=\frac{1}{\left|I(u,v)\right|}\sum_{i\in I(u,v)}C_{P_{i}}^{\left(j\right)}.$$

This averaging ensures that overlapping spatial information is distributed consistently, yielding a coherent contribution map without artificially inflating importance due to redundancy.

**Figure 15:**
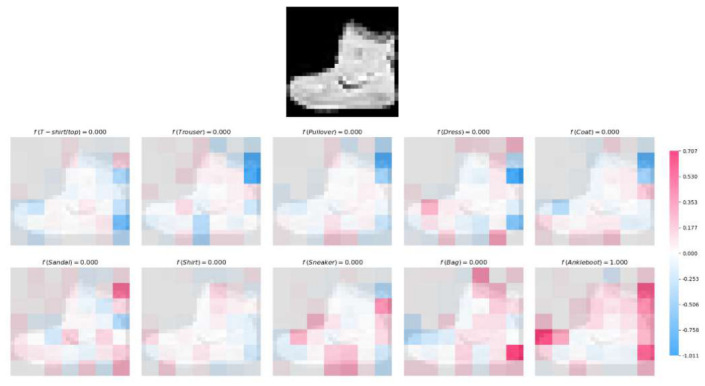
Illustrative visualization of patch-level contributions for a test image classified as *Ankle Boot* using the PP-based architecture. The color scale represents the contribution of each patch to the predicted class, with red indicating positive influence and blue indicating negative influence. For visualization purposes, patches of size 4×4 are used to provide a finer-grained depiction of localized interactions. This configuration is independent of the patch sizes employed in the experimental evaluation (e.g., λ=14, δ∈{7,14}). Regions with stronger positive intensity indicate areas that contribute more strongly to the predicted class, while negative contributions highlight regions that oppose the classification.

Building upon the above formulation, we implement a visual interpretability strategy in which the contribution of each image patch to the prediction of a given class is represented using a continuous color scale. Specifically, contributions are encoded from blue to red, where blue indicates a negative influence and red indicates a positive influence on the class prediction. This visualization provides an intuitive and spatially grounded interpretation of the model’s decision-making process, highlighting the most influential regions of the input image concerning each class. Figure 15 illustrates the interpretability of the prediction generated by our PP-based architecture for a representative test image from the Fashion MNIST dataset, corresponding to the “Ankle Boot” category. The model processes the input image to compute class-specific predictions. Leveraging the polynomial representation of each image patch, we quantify its contribution to the predicted class scores using a continuous color scale (as described earlier, blue and red denote negative and positive influence, respectively). Notably, the patches exhibiting the most intense red coloration correspond to regions strongly supporting the “Ankle Boot” classification. Additionally, the visualization reveals that other categories, such as “Sandal” and “Sneaker,” also receive significant contributions from overlapping or adjacent image regions, underscoring the shared visual features among these classes.

Unlike MLPs and CNNs, whose interpretability typically relies on external approximation techniques, the PP-based architecture admits an intrinsic and analytically tractable decomposition of class predictions. Contributions arise directly from model coefficients and structural connections, providing a transparent link between learned parameters and spatial decision evidence.

### 4.4. Natural Language Processing

**Figure 16:**
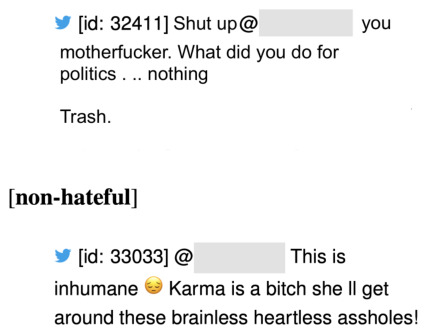
Examples of tweets used in *Subtask A* of SemEval-2019 Task 5, which consists of binary classification of texts as *hateful* (label 1) or *non-hateful* (label 0). Extracted from the original publication: *SemEval-2019 Task 5: Multilingual Detection of Hate Speech Against Immigrants and Women in Twitter* [25].

To further illustrate the versatility of the proposed PPs, we extended its application to a Natural Language Processing (NLP) task. Specifically, we evaluated its performance on the SemEval-2019 Task 5: *Multilingual Detection of Hate Speech Against Immigrants and Women in Twitter* [25], a benchmark designed to assess models’ ability to identify offensive content across different languages and sociocultural contexts. The task addresses the detection of hate speech targeting two specific groups: immigrants and women. It is formulated as a supervised text classification problem using content from Twitter, and is divided into two subtasks. *Subtask A* involves binary classification, where each tweet is labeled *hateful* or *non-hateful*, with hate explicitly directed at one of the two target groups. *Subtask B* requires a more fine-grained analysis, aiming to determine (i) whether the hate speech is directed at an individual or a group, and (ii) whether the language used is aggressive or incites harmful behavior. Here, we focus on *Subtask A*, which provides a suitable testbed for assessing the effectiveness of the proposed PPs. Examples of the instances included in this dataset are shown in Figure 16.

#### 4.4.1. Architecture

**Figure 17:**
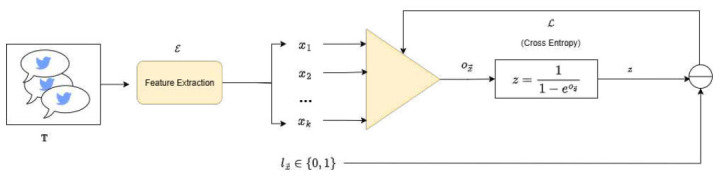
Architecture of PP model for text classification. The input text in T is encoded through a feature extraction function $E:T\to R^{k}$. The resulting feature vectors are then processed by the PP architecture to perform binary classification. The overall workflow parallels the one described in Section 4.1.1, with adaptations to handle textual data.

SemEval-2019 *Subtask A* can be formally regarded as a binary classification task, analogous to the problem delineated in Section 4.1. Since the PP-based architecture for binary classification and its operational dynamics have already been discussed in detail (see Section 4.1.1 and Figure 2), the focus here is placed on the encoding strategy of the input within a model based on our proposal. Figure 17 illustrates the process by which the input text, denoted as the set $T=\{t_{1},t_{2},\ldots ,t_{m}\}$, is transformed into a continuous representation through a feature extraction function:

$$E:T⟶R^{k}\;,$$

where each textual unit $t_{i}\in T$ is mapped to a *k*-dimensional embedding vector ${\overset{\to }{x}}_{i}\in R^{k}$. These embeddings, which capture semantic and syntactic regularities of the input, are then concatenated into the input matrix $X\in R^{m\times k}$ that feeds the PP. Once encoded, the subsequent polynomial transformations and classification layers follow the same computational pipeline described in Section 4.1.1.

The original input T, with m=9000 texts, was prepared with a simple pre-processing procedure that included: apostrophe normalization, URL removal, mention removal, homogenization of consecutive spaces, and hashtag splitting (e.g., #Tag1Tag2 breaks down into #Tag1 and #Tag2). Texts with atypical lengths (too short and too long) were filtered out. To filter the texts, we consider the length distribution in terms of the number of tokens, leaving only texts within the percentile range (2.5, 97.5), which represents 95.44% of the original data. Filtering helps prevent memory overflow in experiments (caused by excessively long texts) and ensures sufficient features are available during training (which very short texts often lack). We identified a class imbalance within the dataset partitions. Specifically, the training set contained 4954 instances (57.7%) annotated with label 0 and 3636 instances (42.3%) annotated with label 1. Such disparities in class distribution may introduce a bias and compromise the robustness of the resulting models. To address this issue, a balanced dataset was constructed, consisting of 4194 instances with label 0 and 4195 instances with label 1.

The implementation of the encoding function E was based on two complementary approaches: (i) Term Frequency–Inverse Document Frequency (TF–IDF) encoding, performed with the TfidfVectorizer component from the scikit-learn library (v1.7.0) using its default configuration; and (ii) *Weighted Word Embedding Averaging*. Further details of this implementation are as follows:

- From the corpus T, the TfidfVectorizer was trained considering exclusively the vocabulary contained in a pre-trained embedding model (GloVe, 100 dimensions), loaded via the Gensim (v4.3.3) package.
- Relying on the resulting term–document matrix, let $w_{i,j}$ denote the *j*-th token in the *i*-th document, with TF–IDF weight $\tau_{i,j}$, and let ${\overset{\to }{x}}_{w_{j}}\in R^{d}$ be its embedding vector. The weighted embedding of each token is then computed as follows:
  $${\overset{\to }{v}}_{i,j}=\tau_{i,j}\cdot {\overset{\to }{x}}_{w_{j}}\;.$$
- Subsequently, the document-level embedding for the *i*-th document is defined as the normalized weighted average:
  $${\overset{\to }{x}}_{i}=\frac{\sum_{j=1}^{n_{i}}\tau_{i,j}\cdot {\overset{\to }{x}}_{w_{j}}}{\sum_{j=1}^{n_{i}}\tau_{i,j}}\;,$$
  where $n_{i}$ denotes the number of tokens in the *i*-th document.

For each document (tweet) in T, the formulation yields a dense semantic representation ${\overset{\to }{x}}_{i}\in R^{k}$, which effectively captures the relative importance of tokens while attenuating the influence of rare or noisy terms. Collectively, these representations were assembled into a matrix $X\in R^{\left|T\right|\times k}$, where each row corresponds to the embedding of a document in T. This matrix subsequently served as input to the PP. Specifically, each tweet vector $\overset{\to }{x}\in X$ is propagated through the binary polynomial network, where the processing unit $u(\overset{\to }{x})$ computes a non-linear transformation of the input. The resulting activation $o_{\overset{\to }{x}}$ is subsequently passed through a sigmoid function $\sigma (o_{\overset{\to }{x}})=z$, which yields the estimated probability of the tweet being hateful. The model parameters, represented by the network’s coefficients, are optimized via gradient-based learning, guided by the binary cross-entropy loss function.

#### 4.4.2. Evaluation

To evaluate the effectiveness of the proposed PP model, we conducted experiments with two configurations, corresponding to polynomial degrees d=2 and d=3. The performance of these configurations was compared against the following reference approaches from the original SemEval-2019 Task 5 (No methodological details were reported for the SemEval-2019 second-place approach):

- The *SemEval Baseline*, based on a linear SVM trained on a TF-IDF representation, with hyperparameters set to the default configuration of the scikit-learn (v1.7.0) Python (v3.13.5) library.
- The *SemEval Winner*, also relying on SVM but using an RBF kernel and leveraging *Google’s Universal Sentence Encoder* to obtain sentence-level embeddings, training solely on the given dataset.
- The *SemEval Third-place*, consisting of a stacked *Bidirectional Gated Recurrent Unit* (BiGRU) architecture, using *fastText* word embeddings as input features.

Table 4 reports the results for binary hate speech classification under both unbalanced (original data distribution) and balanced training conditions.

When trained on the original, unbalanced dataset, PP models achieve moderate performance levels, reflecting the inherent class imbalance of the task. Under balanced training conditions, F1-scores increase substantially, illustrating the sensitivity of polynomial models to class distribution and highlighting their feasibility in controlled settings.

It is important to emphasize that the balanced experiments involve dataset modifications (length filtering and class rebalancing). Therefore, these results are not directly comparable to leaderboard systems trained under the official SemEval data distribution. Comparisons with the baseline, winner, and third-place systems should be interpreted descriptively rather than as strict performance equivalence.

We emphasize that the use of TF–IDF with PP is deliberate and theoretically motivated. Since PP operates directly on explicit feature coordinates, pairing it with TF–IDF ensures coefficient-level interpretability: each learned weight corresponds to a transparent and linguistically meaningful term or interaction. This property would not be preserved under dense learned embeddings.

Under controlled balanced conditions, PPs achieved competitive F1-scores while maintaining explicit polynomial parameterization. These findings suggest that bounded-degree polynomial expansions can operate effectively in hate speech classification when appropriate feature representations and class distributions are considered.

#### 4.4.3. Interpretability

In this setting, interpretability cannot be achieved directly by examining the polynomial coefficients, since the input to the PP consists of embeddings that synthesize token-level information without providing explicit correspondence to the continuous values of the embedding dimensions. To address this limitation, we assess individual tokens’ influence on the model’s predictions. Specifically, each token *t* in the input text is evaluated through a token-wise contribution analysis:

1. For each token *t*, we retrieve its associated TF–IDF weight $\tau_{t}$ and its corresponding GloVe embedding vector $e_{t}$.
2. The contribution of token *t* is isolated by removing its scaled embedding $\tau_{t}e_{t}$ from the overall embedding sum of the input, thereby yielding a modified representation ${\overset{\to }{x}}^{\left(-t\right)}$ that excludes the influence of *t*.
3. The modified embedding ${\overset{\to }{x}}^{\left(-t\right)}$ is then propagated through the trained model, yielding the prediction y^(−t), which reflects the model’s output in the absence of token *t*.
4. Finally, the contribution of token *t* is quantified as the difference between the baseline prediction y^, obtained using the full input, and the counterfactual prediction y^(−t), obtained after removing *t*. Such a contribution can be expressed as follows:
  C(t)=y^−y^(−t).

**Figure 18:**
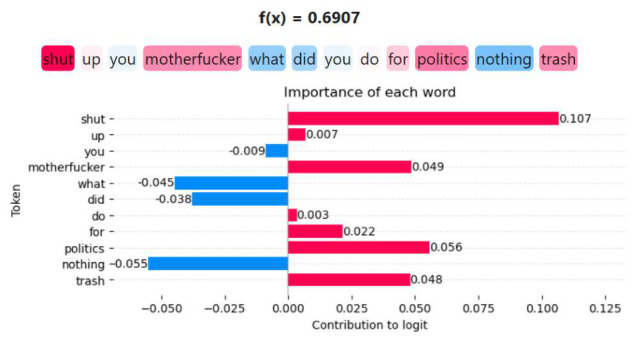
Visualization of token-wise contributions to the prediction of the PP model. Tokens in red denote positive contributions (increasing the probability of hate speech), whereas tokens in blue denote negative contributions (reducing this probability).

The above procedure quantifies the impact of each token on the final prediction, providing an interpretable measure of feature importance at the token level. Figure 18 illustrates an example extracted from the corpus, where a sentence is pre-processed and transformed into an embedding vector $\overset{\to }{x}$, subsequently forwarded to the PP model. The model produces a prediction value f(x→)=y^, which denotes the probability of the sentence being classified as hate speech. From this prediction, the contribution of each token in the sentence is computed, enabling the identification of the terms that exert the greatest influence on the model’s decision. In the visualization, tokens shown in red indicate a positive contribution toward the hate speech prediction, whereas tokens in blue represent a negative contribution. We observe that tokens such as *shut*, *motherfucker*, *politics*, and *trash* exert a strong influence on the model’s decision, highlighting their critical role in driving the prediction.

## 5. Limitations and Scalability Considerations

Explicit polynomial feature expansions inherently suffer from combinatorial growth in the number of coefficients as a function of input dimensionality and polynomial degree. This effect is clearly observed in the global (PP-Flat) configuration, where the number of monomial terms increases rapidly, leading to substantial memory and computational requirements.

The proposed formulation does not eliminate this fundamental limitation. Instead, it mitigates its impact through structured design choices, such as bounding the polynomial degree and restricting interactions to localized subsets of the input (e.g., patch-based decompositions in image data). These strategies reduce the effective dimensionality of the expansion and make training feasible in moderate settings, but the underlying exponential scaling with feature count and degree remains.

As a result, the direct application of global polynomial expansions becomes impractical for high-dimensional inputs without additional constraints. Future extensions may benefit from incorporating structured sparsity, feature selection mechanisms, or regularization techniques to control the growth of coefficients and improve scalability.

The present empirical evaluation should be interpreted as a set of representative case studies rather than a comprehensive benchmarking effort across all data modalities. In particular, the image experiments are currently restricted to single-channel inputs (e.g., Fashion-MNIST). The extension of spatial polynomial constructions to multi-channel inputs such as RGB images has not yet been implemented in the present framework. Consequently, higher-variance benchmarks such as CIFAR-10/100 were not included in this study. Extending the architecture to multi-channel image representations and evaluating performance on additional real-world tabular multiclass datasets constitute important directions for future research.

## 6. Conclusions

This work explored a structured formulation of polynomial-based models through the introduction of bounded-degree, explicitly constructed feature mappings combined with linear parameterization. This study revisits explicit polynomial expansions under a controlled and structured setting, with the goal of examining their behavior across different data modalities and architectural configurations. The empirical results indicate that, within the evaluated settings, localized polynomial constructions can provide a viable alternative for modeling interactions in classification tasks. In particular, the use of bounded-degree expansions and spatially constrained interactions enables tractable implementations that balance expressiveness and computational cost. Across the considered experiments, these models exhibit competitive behavior relative to the selected baselines under comparable experimental conditions while maintaining an explicit representation in which learned coefficients can be directly associated with specific feature interactions.

An important aspect of the proposed formulation lies in its structural transparency. By explicitly encoding interactions through polynomial terms, the model allows for direct inspection of individual contributions, which may be useful in contexts where interpretability and analytical tractability are relevant. At the same time, this study highlights the practical trade-offs inherent to explicit polynomial representations, particularly regarding scalability and coefficient growth, reinforcing the need for structured design choices such as locality and bounded degree.

Overall, the results suggest that structured polynomial constructions constitute a feasible and flexible modeling approach under controlled configurations, offering a complementary perspective to implicit feature learning strategies in contemporary machine learning.

## Figures and Tables

**Table 1:** Comparative performance and model complexity for the binary classification problem. Unlike the PP or MLP, the SVM does not learn an explicit finite-dimensional weight vector; the effective model complexity depends on the number of support vectors.

Model	Configuration	Test Accuracy	Parameters
PP	Degree d=1	0.8850	3
PP	Degree d=2	0.8783	6
PP	Degree d=3	0.9967	10
SVM	RBF kernel	0.9983	Implicit
MLP	1 layer (2)	0.8850	9
MLP	1 layer (4)	0.8883	17
MLP	1 layer (8)	0.8983	33
MLP	1 layer (16)	0.9967	65

**Table 2:** Multiclass Ablation Results. Comparison of Polynomial Perceptrons (PP) of increasing degree against SVM (RBF) and shallow MLP architectures.

Model	Configuration	Test Accuracy	Parameters
PP	Degree 1	0.8333	9
PP	Degree 2	0.8567	18
PP	Degree 3	0.8667	30
PP	Degree 4	0.8700	45
SVM	RBF kernel	0.8667	implicit
MLP	1-layer (4 neurons)	0.8617	27
MLP	1-layer (8 neurons)	0.8733	51
MLP	1-layer (16 neurons)	0.8700	99

**Table 3:** Test-set accuracy under approximate capacity normalization.

Model	Test Accuracy	Parameters	Structure
PP-Flat	88.9%	3,085,050	Global Polynomial
PP-Local (δ=14)	92.7%	78,062	Local Polynomial
PP-Local (δ=7)	94.1%	175,627	Overlapping Local Polynomial
MLP-100	88.3%	79,510	Dense
MLP-224	89.1%	178,090	Dense
CNN-Low	90.4%	50,186	Convolutional
CNN-Medium	92.0%	230,218	Convolutional

**Table 4:** Results for binary hate speech classification. Comparison of the proposed PP model (with d∈{2,3}) against the SemEval-2019 Subtask A baseline, winner, and third-place approaches. Measures reported include Accuracy, F1-score, Precision, Recall, and number of model parameters.

	Test				
	Accuracy	F1-Score	Precision	Recall	Parameters
SemEval Baseline (SVM, TF-IDF)	−	0.4510	−	−	−
SemEval Winner (Google, SVM-RBF kernel)	−	0.6510	−	−	−
SemEval Third-place (fastText, BiGRU)	−	0.5350	−	−	−
Polynomial (*tfidf, unbalanced, deg* = 2)	0.5601	0.3071	0.4609	0.2302	1326
Polynomial (*tfidf, balanced, deg* = 2)	0.5318	0.4676	0.5419	0.4111	1326
Polynomial (*tfidf, unbalanced, deg* = 3)	0.5609	0.2071	0.4396	0.1355	23,426
Polynomial (*tfidf, balanced, deg* = 3)	0.5405	0.4914	0.5502	0.4440	23,426
Polynomial (*tfidf+GloVe, unbalanced, deg* = 2)	0.5856	0.6349	0.5063	0.8511	5151
Polynomial (*tfidf+GloVe, balanced, deg* = 2)	0.7142	0.7115	0.7185	0.7046	5151
Polynomial (*tfidf+GloVe, unbalanced, deg* = 3)	0.5703	0.6254	0.4955	0.8476	176,851
Polynomial (*tfidf+GloVe, balanced, deg* = 3)	0.6998	0.7006	0.6986	0.7027	176,851

## Data Availability

The source code and experimental materials supporting this study are publicly available at: https://doi.org/10.6084/m9.figshare.31983450. The repository includes the full implementation of the proposed Polynomial Perceptron models, along with notebooks required to reproduce all experiments. The datasets used in this study are publicly available from their official sources and are not redistributed as part of this work: Fashion-MNIST dataset (accessed on 10 April 2026): https://github.com/zalandoresearch/fashion-mnist; and SemEval-2019 Task 5 (accessed on 10 April 2026): https://huggingface.co/datasets/valeriobasile/HatEval. Instructions for downloading and preparing these datasets are provided in the repository. Users are required to download the datasets from the official sources and place them in the designated data/directory prior to running the experiments. Synthetic datasets used in tabular experiments are generated directly within the provided notebooks. All materials are provided to ensure full reproducibility of the reported results.
